# Cross-domain zero-shot semantic segmentation for unstructured environments via EVA-CLIP model, ensemble prompt engineering, and optimized text-image matching

**DOI:** 10.1371/journal.pone.0352325

**Published:** 2026-06-26

**Authors:** Nana Zhou, Xianhua Zhao, Fengjun Zhou, Ji Li, Xiuming Yue

**Affiliations:** 1 School of Computer Science, Shandong Xiehe University, Shandong, China; 2 Jinan Energy Investment Holding Group Co., Ltd., Shandong, China; State Grid Corporation of China, CHINA

## Abstract

Semantic segmentation provides essential scene understanding for unmanned ground vehicles to identify obstacles and plan paths in unstructured environments. Nevertheless, existing methodologies tailored for these settings typically necessitate linear probing or fine-tuning to accommodate novel scenarios, thereby suffering from a deficiency in zero-shot transferability. In response to this limitation, our study introduces a novel framework designed for robust zero-shot transfer in unstructured domains, capitalizing on the superior visual-linguistic alignment capabilities of the EVA-CLIP architecture. To augment segmentation precision, we initially utilize deep prompt tuning to adapt the visual feature extraction efficacy of the EVA-CLIP image encoder to unstructured terrain features. This strategy not only bolsters adaptability to irregular environments but also preserves the intrinsic zero-shot proficiency of the underlying model. Concurrently, we devise an ensemble prompt engineering scheme customized for unstructured settings to further elevate segmentation outcomes. Moreover, the framework optimizes the correspondence between text and images by integrating global and local representations from the respective encoders, thereby maximizing cross-modal alignment for superior segmentation. Empirical evaluations indicate that our methodology surpasses contemporary state-of-the-art techniques, yielding an increase in *mIoU* ranging from 1.2% to 43.9% on the Robot Unstructured Ground Driving (RUGD) benchmark. Furthermore, evaluations on the Rellis-3D dataset reveal that the model’s cross-domain zero-shot performance rivals that of supervised fine-tuning approaches, demonstrating robust generalization to previously unseen semantic classes.

## 1. Introduction

The rapid evolution of Unmanned Ground Vehicles (UGVs) has catalyzed a surge in research dedicated to outdoor autonomous mobility, specifically within complex, unstructured domains [1–3]. This operational scope encompasses the traversal of rugged topography and the management of intricate scenarios prevalent in sectors including precision agriculture, disaster relief operations, mining, and ecological surveillance.

Within the specific domain of UGV mobility in non-standardized terrains, visual semantic segmentation constitutes a pivotal technological pillar [4,5]. In contrast to conventional navigational frameworks dependent exclusively on geometric data or pre-defined trajectories, this approach endows systems with the capacity for holistic environmental interpretation [6]. Through the granular analysis of visual input and the classification of individual pixels into distinct classes—such as vegetation, rigid structures, or terrain—UGVs achieve a sophisticated level of scene comprehension [7]. This augmented perceptual acuity facilitates superior decision-making, allowing the UGV to reliably discriminate between navigable paths and obstructive features [8,9].

Nevertheless, a prominent bottleneck in contemporary segmentation frameworks for unstructured settings is their reliance on retraining phases, such as linear probing or fine-tuning, when deployed in novel environments [10]. This dependency stems from the discrepancies in taxonomic annotations across diverse datasets, which preclude the immediate, zero-shot transferability of pre-trained architectures. Consequently, adapting to each unique operational context mandates resource-intensive and time-consuming model customization [11].

**Fig 1:**
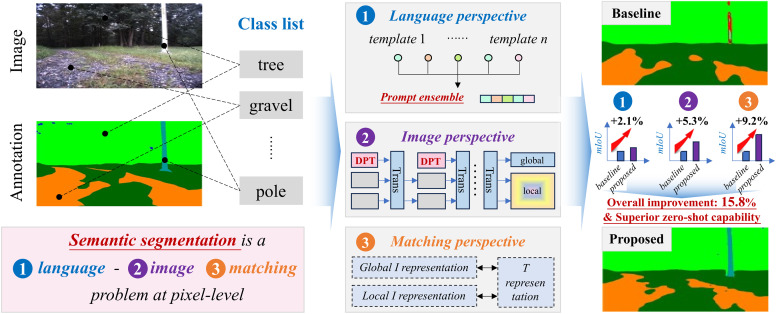
Innovation of the proposed semantic segmentation method.

The Contrastive Language-Image Pre-training (CLIP) model has emerged as a transformative force in computer vision, distinguished by its formidable zero-shot potential [12]. Its architecture facilitates adaptation to unseen scenarios without supplementary training by forging a robust semantic alignment between linguistic prompts and visual content [13]. By merely supplying textual definitions of target categories, CLIP can successfully classify visual data, even when those categories were absent from its primary training corpus [14]. Advancing this foundation, EVA-CLIP proposes a streamlined and potent pre-training methodology [15]. Its primary innovation involves initializing the vision encoder via a massive masked image modeling precursor (e.g., EVA) rather than stochastic initialization. This strategy permits the assimilation of comprehensive, transfer-ready visual features from vast unlabeled datasets prior to engaging in cross-modal synchronization. As a result, EVA-CLIP attains superior efficacy with reduced reliance on labeled image-text pairs, exhibiting exceptional scalability and generalization across diverse downstream tasks. In this study, we frame semantic segmentation in unstructured terrains as a text-image alignment challenge, aiming to exploit the zero-shot proficiency of EVA-CLIP to bolster generalization performance. We enhance the model’s cross-modal matching competence by refining both visual and textual dimensions, independently and synergistically, as illustrated in Fig 1. The primary contributions of our approach are threefold.

Initially, addressing the linguistic dimension, we implement an Ensemble Prompt Engineering (EPE) strategy [16] that is explicitly calibrated for complex, unstructured terrains to elevate segmentation efficacy. Empirical evidence suggests that the integration of such ensemble methodologies yields substantial enhancements across key evaluation metrics.

Conversely, regarding the visual domain, the Deep Prompt Tuning (DPT) mechanism [17] is utilized to fortify the feature extraction proficiency of the EVA-CLIP vision encoder. Our analysis reveals that DPT not only refines the framework’s supervised adaptation to unstructured settings but also safeguards the intrinsic zero-shot potential characteristic of the underlying EVA-CLIP architecture.

Furthermore, we execute a Text-Image Matching Optimization (TIMO) by fusing global and local feature representations derived from both the EVA-CLIP visual and textual encoders. By combining global and local features, this alignment ensures that the model matches text labels with both the overall scene context and specific object details, improving segmentation accuracy.

Independently, these three architectural innovations yield performance increments of 2.1%, 5.3%, and 9.2% in terms of mean Intersection over Union (*mIoU*), respectively. When integrated, these components culminate in a total performance improvement of 15.8% on the Robot Unstructured Ground Driving (RUGD) dataset. The near-additive nature of these improvements—where the integrated performance increase of 15.8% closely approximates the theoretical sum of individual increments 16.6%—underscores the architectural orthogonality and functional complementarity of the three proposed modules. This phenomenon highlights the specific methodological gap our framework bridges: the inherent inability of standard CLIP-based models to simultaneously reconcile linguistic ambiguity, visual domain shift, and the context-granularity mismatch in unstructured terrains. Specifically, while EPE mitigates bias within the linguistic embedding space by aggregating multifaceted textual perspectives, and DPT calibrates the vision encoder to the irregular morphologies of organic objects without sacrificing the model’s intrinsic zero-shot knowledge, TIMO addresses the interaction bottleneck by fusing global scene-level descriptors with local patch representations. By independently yet synergistically optimizing the input text, the visual feature extraction, and the cross-modal matching mechanism, the proposed framework ensures that the enhancements in each modality do not suffer from information redundancy. Instead, they form a cohesive synchronization that is uniquely robust for dense, pixel-level prediction in stochastic, non-geometric unstructured environments. Additionally, owing to the integration of the EVA-CLIP backbone, the model following supervised adaptation on RUGD exhibits exceptional zero-shot transferability when applied to the unobserved Rellis-3D dataset.

The subsequent organization of this manuscript is as follows: Section 2 provides a comprehensive review of pertinent literature concerning semantic segmentation, with a particular emphasis on applications in unstructured environments. Section 3 elucidates the proposed methodology in depth, highlighting the three novel techniques responsible for the marked increase in segmentation accuracy. Section 4 validates the proposed framework through rigorous testing on two distinct unstructured datasets—specifically, supervised evaluation on RUGD and zero-shot transfer evaluation on Rellis-3D—and Section 5 offers concluding remarks.

## 2. Related work

The research of semantic segmentation has undergone a fundamental transition towards deep learning methodologies [18]. Initial investigations, which depended heavily on manual feature engineering and graphical models [19], frequently failed to comprehend the intricacies of complex scenes. The advent of fully convolutional networks in 2014 represented a watershed moment, validating the efficacy of end-to-end training protocols for dense, pixel-level prediction [20,21]. Following this, the U-net framework, characterized by its encoder-decoder configuration, significantly augmented performance by synthesizing abstract semantic data with granular spatial details [22]. Contemporary scholarship investigates further enhancements, such as the utilization of dilated convolutions to expand receptive fields [23], and the deployment of attention mechanisms to prioritize salient image regions [24]. Moreover, Transformer architectures, originally ubiquitous in natural language processing, are penetrating the segmentation domain, opening novel pathways for superior image comprehension and robust analysis [25–27].

Despite substantial strides in segmentation algorithms, the literature predominantly targets structured domains, such as urban landscapes [28]. Conversely, investigations dedicated to unstructured terrains—including off-road paths and natural environments—remain relatively sparse. This paucity of research constitutes a major impediment to the engineering of resilient perception frameworks for UGVs facing variable illumination, amorphous object morphologies, and chaotic spatial configurations [29]. Addressing this research gap through the development of specialized segmentation frameworks for unstructured environments is essential to ensuring the operational safety and efficiency of UGVs across diverse and unpredictable domains.

Performing semantic segmentation in unstructured contexts involves difficulties distinct from those encountered in controlled environments [30]. The prevalence of organic components, such as vegetation and rugged topography, introduces geometric irregularities that diverge significantly from the standardized objects typical of structured settings [31]. Furthermore, the stochastic layout of natural scenes, devoid of the organized structure inherent to man-made spaces, complicates the model’s ability to acquire robust contextual dependencies. Collectively, these variables precipitate a marked degradation in accuracy when models trained on structured datasets are deployed in the wild.

To mitigate these obstacles in unstructured domains, Guan et al. introduced GA-Nav, utilizing a group-wise attention mechanism to classify terrain traversability levels [32]. Lin et al. explored out-of-domain generalization by embedding semantic-aware normalization and whitening modules into the DDRNet23-slim architecture [33]. Additionally, Fu et al. engineered a density-sensitive nested U-structure specifically for dust segmentation in mining operations, underscoring the domain-specific hurdles within unstructured settings [34]. Nevertheless, current methodologies suffer from a rigid dependency on fine-tuning or linear probing to accommodate novel categories, a process that is both resource-intensive and computationally demanding given the disparity in annotation schemas [35]. Consequently, zero-shot semantic segmentation is of paramount importance for unstructured environments due to the logistical complexities of data annotation in these dynamic settings [36]. In contrast to structured environments where datasets can be extensively annotated, obtaining exhaustive labels for the diverse array of entities encountered in natural settings is often logistically impractical. Cross-domain zero-shot paradigms offer a robust alternative by empowering models to exploit pre-existing semantic knowledge and textual prompts to identify unseen classes. This capability is essential for sustaining robust perception systems in unstructured environments where the emergence of novel obstacles is inevitable.

The CLIP architecture represents a monumental leap in the sphere of multimodal learning. By employing a contrastive learning approach, CLIP aligns images and linguistic descriptions within a shared latent dimensionality [37]. This dual-modality training fosters potent associations between visual and textual features, facilitating task execution beyond the original training distribution. Specifically, EVA-CLIP demonstrates exceptional proficiency in zero-shot inference, executing retrieval and classification tasks without requiring dataset-specific optimization [38]. This aptitude derives from the model’s capacity to transfer learned semantic alignments to novel data, rendering it invaluable for adaptive artificial intelligence systems [39,40]. Accordingly, our objective is to leverage the robust semantic alignment of EVA-CLIP within a framework that undergoes supervised adaptation on source unstructured terrains to achieve zero-shot transferability when deployed in unobserved operational contexts.

The shift toward leveraging massive pre-trained architectures for domain-specific tasks is increasingly driven by the need for data efficiency. For instance, in the energy sector, the PowerMistral framework demonstrates how pre-trained large language models like Mistral-7B can be adapted for few-shot wind power forecasting, significantly reducing the reliance on historical operational data through a two-step fine-tuning process and trend-related textual prompts [41]. This paradigm of real-time, data-driven assessment is further extended by methodologies that utilize state-space mapping and Koopman operator theory to evaluate complex system dynamics. Such approaches prove essential when dealing with incomplete parameters and the need for rapid, model-independent generalization [42]. Moreover, the challenge of maintaining methodological robustness under significant uncertainty—a core hurdle in our study of unstructured terrains—parallels recent developments in multi-integrated energy microgrids, where bi-level hybrid games and two-stage stochastic robust optimization are employed to facilitate structured multi-stage evaluation amidst complex operational couplings [43]. In a similar vein, our framework exploits the intrinsic zero-shot proficiency of the EVA-CLIP architecture. By employing prompt-based adaptation techniques that mirror the pattern-recognition logic used in high-uncertainty time-series tasks, we ensure that the model retains broad generalization capabilities while achieving high-fidelity pixel-level predictions in novel, non-geometric terrains.

## 3. Methodology

Cross-domain zero-shot transfer in semantic segmentation constitutes a formidable frontier within the domain of computer vision. Distinct from conventional segmentation paradigms predicated on supervised learning across a fixed set of labels, this methodology aims to delineate objects corresponding to unseen classes entirely absent from the initial training distribution. To achieve this, the architecture must exhibit a profound comprehension of the intrinsic alignment between visual modalities and semantic concepts, a capability essential for extrapolating learned associations to novel categories using linguistic descriptors.

**Fig 2:**
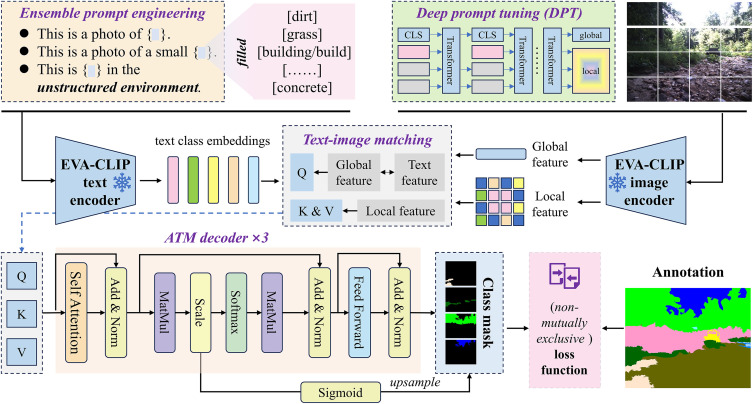
General framework of the proposed cross-domain zero-shot semantic segmentation method for unstructured environments.

The architectural schema of our proposed cross-domain zero-shot framework is illustrated in Fig 2. Structurally, the system is tripartite, consisting of: a textual feature extraction module (positioned at the top-left of Fig 2), a visual feature extraction module (top-right of Fig 2), and a cross-modal alignment mechanism (central and lower sections of Fig 2). Specifically, the textual encoding subsystem integrates Ensemble Prompt Engineering (EPE) with the EVA-CLIP text encoder. Its primary function is to interpret semantic category labels—spanning both those observed during training and novel classes introduced at inference—to synthesize robust textual embeddings. In parallel, the visual encoding subsystem leverages Deep Prompt Tuning (DPT) in conjunction with the EVA-CLIP image encoder to decompose input imagery, thereby yielding hierarchically structured global and local feature representations. Crucially, the internal weights of both the EVA-CLIP text and vision encoders are held static. They are initialized using the pre-trained weights detailed in the original EVA-CLIP study [15] and are excluded from gradient updates throughout the training phase. Finally, the text-image matching component exploits the semantic resonance between linguistic and visual vectors. This alignment facilitates the derivation of dense, pixel-level classifications via an Attention-to-Mask (ATM) decoding head. The resulting predictions are then evaluated against ground truth data via a tailored loss function, which drives the optimization of the trainable network components through error backpropagation.

### 3.1. Textual feature extraction part

In standard semantic segmentation protocols, category labels are typically enumerated as discrete lexical tokens—such as concrete, bushes, trees, or sky—exemplified by the taxonomy of the RUGD dataset. Nevertheless, directly feeding isolated nouns into the EVA-CLIP textual encoder often proves suboptimal. This limitation arises because the model’s pre-training corpus predominantly consists of imagery paired with descriptive natural language sentences rather than solitary keywords.

Therefore, it makes much more sense to use the prompt template like “*A photo of a {class}*.” as the input to the EVA-CLIP text encoder.

To augment semantic segmentation efficacy, we employ a strategy of prompt ensembling that aggregates templates within the embedding domain, as opposed to the probability space. This architectural choice enables the pre-calculation of a mean textual embedding vector, thereby ensuring that the inference latency of the ensemble model remains computationally isomorphic to that of a standard, single-classifier framework.

The precise specifications of the 13 augmented templates utilized are enumerated below:

- A photo of a {*class*} in the unstructured environment.
- A photo of a small {*class*} in the unstructured environment.
- A photo of a medium {*class*} in the unstructured environment.
- A photo of a large {*class*} in the unstructured environment.
- This is a photo of a {*class*}.
- This is a photo of a small {*class*}.
- This is a photo of a medium {*class*}.
- This is a photo of a large {*class*}.
- A {*class*} in the unstructured environment.
- A photo of a {*class*} in the unstructured environment.
- There is a {*class*} in the unstructured environment.
- There is one {*class*} in the unstructured environment.
- This is a {*class*} in the unstructured environment.

The architecture of EVA-CLIP distinguishes itself within the vision-language domain primarily through the robust design of its visual encoding component, which significantly augments the original CLIP framework. Built upon the Vision Transformer (ViT) paradigm, the vision encoder benefits from an initialization strategy utilizing a massive model trained via masked image modeling, as elucidated by [15]. Such a pre-training regimen ensures the acquisition of highly transferable visual features antecedent to any cross-modal synchronization efforts. To maintain architectural parity, the textual encoder adheres to the conventional Transformer methodology. We utilize a baseline configuration consisting of 63M parameters, organized across 12 layers, featuring a channel width of 512 and 8 distinct attention heads. Linguistic inputs are processed via a lower-cased byte pair encoding scheme, drawing from a vocabulary of 49152 tokens [44]. The sequence is bookended by [SOS] and [EOS] markers, where the activation of the final layer corresponding to the [EOS] token serves as the representative feature vector. Subsequent to layer normalization, this vector undergoes linear projection to map it onto the shared multi-modal embedding manifold. The fundamental advancement of this approach stems from the contrastive alignment of the robust, pre-trained ViT backbone with the textual encoder, thereby realizing enhanced computational efficiency and broader generalization capabilities.

### 3.2. Visual feature extraction part

Given the robust feature extraction capabilities inherent in the pre-trained EVA-CLIP vision encoder, our objective is to optimize its applicability to unstructured domains without altering its foundational parameters. To this end, we incorporate the DPT methodology. As a contemporary innovation in model adaptation [17], DPT diverges from conventional fine-tuning—which necessitates extensive weight adjustments—by employing calibrated prompts to steer the model’s inference trajectory. This paradigm confers distinct benefits, most notably a reduction in task-specific parameter overhead and the facilitation of concurrent multi-task deployment on a unified architecture. Furthermore, it yields performance metrics that frequently surpass those achieved via full-model fine-tuning across diverse benchmarks.

In the context of the ViT-based framework underlying the EVA-CLIP vision encoder, let the input embeddings corresponding to the $l^{th}$ Multi-Head Attention (MHA) block be formalized as $\left\{g_{N}^{l},h_{1}^{l},h_{2}^{l},\ldots ,h_{N}^{l}\right\}$. Here, $g^{l}$ symbolizes the embedding for the distinct [CLS] token, while the set of image patch embeddings is defined as $H^{l}=\left\{h_{1}^{l},h_{2}^{l},\ldots ,h_{N}^{l}\right\}$. The DPT mechanism integrates a set of trainable tokens, expressed as $P^{l}=\left\{p_{1}^{l},p_{2}^{l},\ldots ,p_{M}^{l}\right\}$, which are concatenated to the pre-existing token sequence at every layer of the EVA-CLIP vision encoder. Accordingly, the $l^{th}$ MHA unit operates upon this augmented sequence, which amalgamates both visual data and prompt indicators, in the following manner:


$$\begin{array}{c} \left[g^{l},\_,H^{l}\right]={\text{Layer}}^{l}\left(\left[g^{l-1},P^{l-1},H^{l-1}\right]\right) \end{array}$$ (1)

where the output embeddings of $P^{l}$ are discarded (denoted as _) and will not feed into the next layer. Therefore, $P^{l}$ merely acts as a set of learnable parameters to adapt the MHA model.

### 3.3. ATM with TIMO enhancement

The ATM module, originally introduced within the SegViT framework [45], diverges from conventional mask-to-attention paradigms. Rather than adhering to standard protocols, it exploits intrinsic attention dynamics to directly synthesize segmentation masks. The ATM architecture operates by ingesting class-specific embeddings alongside feature maps extracted from the primary ViT backbone. By computing the affinity between these components, the internal attention mechanism effectively delineates image regions associated with distinct semantic categories, subsequently converting these similarity scores into definitive segmentation outputs.

To be precise, let the textual class embedding tokens be defined as $𝒢\in R^{N\times C}$, wherein *N* signifies the total count of semantic classes. Concurrently, the visual embedding tokens are represented by ${ℱ}_{i}\in R^{L\times C}$, with *L* corresponding to the aggregate number of image patches. Given the utilization of a ViT infrastructure, the relationship *L* = *HW*/*P*^2^ holds true, where *H* and *W* denote the vertical and horizontal dimensions of the image, respectively, and *P* indicates the patch resolution. Across both tensors $𝒢\in R^{N\times C}$ and ${ℱ}_{i}\in R^{L\times C}$, the variable *C* encapsulates the channel dimensionality. Following this, each token sequence is subjected to linear projection operations, culminating in the derivation of the query (*Q*), key (*K*), and value (*V*) vectors:


$$\begin{array}{c} \begin{array}{c} Q=\phi_{g}(𝒢)\in R^{N\times C},K=\phi_{k}\left({ℱ}_{i}\right)\in R^{L\times C},V=\phi_{v}\left({ℱ}_{i}\right)\in R^{L\times C} \end{array} \end{array}$$ (2)

Subsequently, the calculation of the affinity matrix correlating the query vectors with the key representations is executed. In accordance with the foundational principles of scaled dot-product attention, the derivation of both the correspondence scores and the resultant attention distribution proceeds via the formulation below:


$$\begin{array}{c} S(Q,K)=\frac{QK^{T}}{\sqrt{d_{k}}}\in R^{N\times L} \end{array}$$ (3)

$$\begin{array}{c} \text{Attention}\left(𝒢,{ℱ}_{i}\right)=\text{Softmax}(S(Q,K))V\in R^{N\times C} \end{array}$$ (4)

where $\sqrt{d_{k}}$ is a scaling factor with $d_{k}$ corresponds to the dimensionality of the keys. The shape of the similarity map *S*(*Q*,*K*) is contingent upon the lengths of the two token sequences, *N* and *L*. Specifically, the attention mechanism refines the textual embeddings 𝒢 through a weighted aggregation of the visual feature vectors *V*. The corresponding attention weights are derived by applying a softmax operation to the affinity matrix *S* across the *L* tokens, thereby capturing the spatial relevance of each image patch to the respective semantic classes.

Following this, a computationally efficient component is deployed to immediately synthesize the semantic outputs. In this context, 𝒢 embodies the category-specific embeddings requisite for the segmentation objective, whereas ${ℱ}_{i}$ corresponds to the latent representations extracted from the *i*-th layer of the ViT model. Crucially, every token residing in 𝒢 is intrinsically linked to a semantic mask that characterizes the class-specific prediction. The mathematical formulation governing the generation of this mask is presented below:


$$\begin{array}{c} \text{Mask}\left(𝒢,{ℱ}_{i}\right)=\text{Sigmoid}(S(Q,K))\in R^{N\times L} \end{array}$$ (5)

The resulting mask tensors possess an initial dimensionality of N×L, which are subsequently reconfigured into a spatial tensor format of N×H/P×W/P. To restore the native resolution of N×H×W, a transposed convolution operation is applied for upsampling, wherein each individual channel aligns with a distinct semantic category defined in 𝒢.

An analysis of the foundational ATM framework—with particular reference to Eq. (2)—reveals that the mechanism exclusively utilizes textual embeddings as the query vector, while assigning patch-level visual embeddings to the roles of key and value. Nevertheless, it is imperative to acknowledge that the EVA-CLIP vision encoder yields more than merely local patch representations. It concurrently produces a global visual descriptor, denoted as $g^{l}$, derived from the [CLS] token. Conventional ATM implementations neglect this holistic visual context, a substantial oversight that attenuates the efficacy of cross-modal alignment. To mitigate this deficiency, we introduce the novel TIMO architecture. In this framework, the query component detailed in Eq. (2) is substituted with a hybrid representation that amalgamates both the textual embedding and the global visual feature, as demonstrated below:


$$\begin{array}{c} \begin{array}{c} Q_{c}=\phi_{gc}(𝒢)\in R^{N\times C},K_{c}=\phi_{kc}\left(g^{l}\right)\in R^{1\times C},V_{c}=\phi_{vc}(𝒢)\in R^{N\times C} \end{array} \end{array}$$ (6)

$$\begin{array}{c} Q=\text{Softmax}\left(S\left(Q_{c},K_{c}\right)\right)V_{c}\in R^{N\times C} \end{array}$$ (7)

The preceding mathematical formulation illustrates that, within the TIMO paradigm, the reformulated query quantifies the divergence between holistic visual descriptors and linguistic embeddings. This evaluation occurs within a linearly projected manifold governed by the mapping functions $\phi_{gc}$, $\phi_{kc}$, and $\phi_{vc}$. In this specific configuration, the textual category tokens function dually as the query and value vectors within the attention module, whereas the global visual attribute assumes the role of the key.

Within the operational scope of the attention mechanism, individual textual class tokens engage with the global visual feature to derive an affinity metric. Consequently, every row in the resultant matrix signifies the degree of correspondence between the global visual context and a discrete semantic class token. The integration of linear projections $\phi_{gc}$, $\phi_{kc}$, and $\phi_{vc}$ substantially bolsters the plasticity and representational power of the TIMO procedure, facilitating more nuanced dynamic interplay between textual and visual modalities.

Through the assimilation of these linear transformations, the TIMO architecture effectively synthesizes text-image correspondences across both global and local scales. The attention mechanism synchronizes global visual data with textual category embeddings, a process that operates concurrently with—and is reinforced by—the intrinsic local matching proficiency of the standard ATM module. Ultimately, this methodology elevates semantic segmentation accuracy by exploiting the synergistic benefits of cross-modal alignment at multiple feature levels, culminating in a more profound interpretation of the input data.

### 3.4 Loss function

Conventionally, semantic segmentation is conceptualized as a per-pixel multi-class classification problem, which relies on the *Softmax* function to derive posterior probabilities in conjunction with cross-entropy as a mutually exclusive objective function. Nevertheless, the rigid assumption inherent in *Softmax*—that a single pixel is exclusively associated with one category—often leads to suboptimal calibration of logits, particularly when the model attempts to generalize to novel, unobserved classes during the inference phase.

To ameliorate these deficiencies, the proposed methodology adopts a non-mutually exclusive loss paradigm. Specifically, we integrate sigmoid activation with Weighted Cross-Entropy (WCE) loss to guarantee that the segmentation inference for each category is generated independently. The deployment of WCE loss proves advantageous in rectifying class imbalance issues by modulating the significance of distinct categories proportional to their prevalence or relevance within the training corpus. Consequently, this strategy bolsters the recognition accuracy of underrepresented classes, fostering a more equitable and precise segmentation landscape across the entire spectrum of categories. Complementing this, the Dice loss is incorporated as an auxiliary objective to further refine the model’s predictive fidelity.

Mathematically, the WCE loss function is formally articulated as:


$$\begin{array}{c} \begin{array}{c} {ℒ}_{WCE}=-\frac{1}{hw}\sum_{i=1}^{hw}\sum_{c=1}^{C}w_{c}[y_{i,c}\log\left({\hat{y}}_{i,c}\right)+\left(1-y_{i,c}\right)\log\left(1-{\hat{y}}_{i,c}\right)] \end{array} \end{array}$$ (8)

where *C* denotes the total number of semantic categories, and $w_{c}$ represents the weighting factor assigned to class *c*. The variables $y_{i,c}$ and ${\hat{y}}_{i,c}$ signify the one-hot encoded ground-truth label and the predicted posterior probability, respectively, for pixel *i* with respect to category *c*.

For the Dice loss, it can be written as follows:


$$\begin{array}{c} {ℒ}_{\text{dice }}=1-\frac{2\sum_{i=1}^{hw}y_{i}\hat{y_{i}}}{\sum_{i=1}^{hw}y_{i}^{2}+\sum_{i=1}^{hw}\hat{y_{i}^{2}}} \end{array}$$ (9)

The total loss function is a weighted combination of WCE loss and Dice loss:


$$\begin{array}{c} ℒ=\alpha \cdot {ℒ}_{\text{WCE}}+\beta \cdot {ℒ}_{\text{dice }} \end{array}$$ (10)

where α and β are coefficients to balance the contributions of WCE loss and Dice loss.

## 4. Validation and analysis

The experimental evaluation begins by characterizing the two specific unstructured environment benchmarks leveraged in this study: the RUGD and Rellis-3D datasets. Following this, we establish the quantitative protocols and metrics utilized to assess semantic segmentation fidelity. Lastly, we present a rigorous ablation analysis alongside a comparative assessment against prevailing state-of-the-art techniques to empirically validate the advanced capabilities of the suggested framework.

### 4.1. Datasets

**Fig 3:**
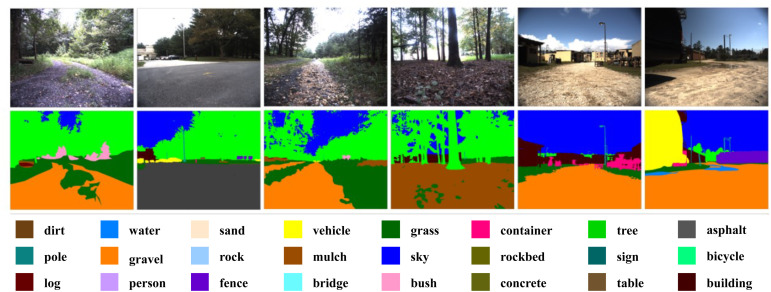
Ground truth annotations examples provided in the RUGD dataset.

**Fig 4:**
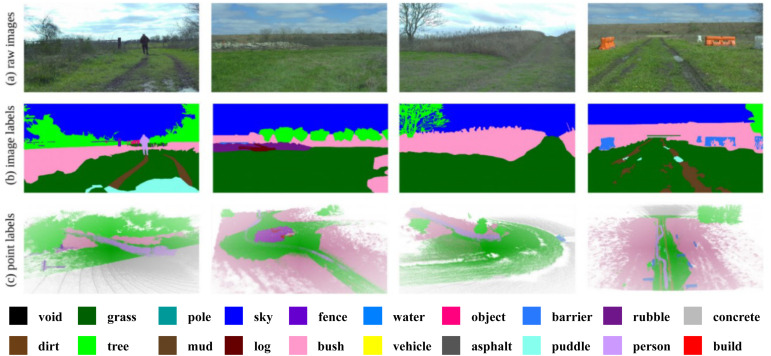
Ground truth annotations examples provided in the Rellis-3D dataset.

Contemporary developments in semantic segmentation have yielded substantial precision gains across standard object recognition benchmarks, including PASCAL VOC [46], COCO [47], and ADE20K [48], in addition to autonomous driving-centric archives like Cityscape [49] and KITTI [50]. Nevertheless, a conspicuous deficiency persists in the literature regarding recognition and segmentation within unstructured, off-road domains, which is a prerequisite for robust autonomous navigation. Sensory perception in such non-standardized environments is fraught with difficulties, primarily stemming from the ambiguity of boundaries delineating various classes, such as distinguishing between aqueous surfaces and asphalt. To bridge this gap, the community has introduced specialized datasets such as RUGD [51] and Rellis-3D [52], expressly curated for the demands of off-road semantic analysis. The RUGD corpus captures a wide array of scenarios—ranging from trails and creeks to parklands and rural settlements—providing high-resolution, pixel-level semantic annotations. Building upon this foundation, the Rellis-3D dataset broadens the operational horizon by incorporating distinct terrain morphologies, thereby diversifying the available resources for domain-specific research. A comparative taxonomy of the classes present in both repositories is detailed in Table 1, while representative visual samples are depicted in Fig 3 and Fig 4. For the purpose of this investigation, imagery and corresponding ground-truth labels from both RUGD and Rellis-3D are employed to substantiate the efficacy of our proposed framework. Specifically, the model undergoes supervised training on the RUGD dataset, followed by an evaluation of its zero-shot transfer capabilities on the entirely unobserved Rellis-3D benchmark. To guarantee an equitable comparative analysis, the stratification of the RUGD dataset into training, testing, and validation subsets adheres strictly to the protocol established in Ref. [32].

**Table 1:** Comparison of annotations between RUGD and Rellis-3D datasets.

	RUGD dataset	Rellis-3D dataset
**Common**	void, dirt, grass, tree,	
**annotations**	pole, water, sky, vehicle, asphalt,	
	building/build, log, person,	
	fence, bush, concrete	
**Different**	sand, container/generic-object,	
**annotations**	gravel, mulch, rock-bed,	object, barrier,
	bicycle, sign, rock,	puddle, mud, rubble
	bridge, picnic-table	

In specific operational contexts—most notably those involving vehicular mobility through unstructured terrains—the primary objective of semantic segmentation often pivots from the exhaustive categorization of discrete entities (e.g., individual flora) to the binary delineation of traversable versus non-traversable zones. Consequently, adhering to the methodology established in Ref. [32], we execute a reclassification of the taxonomic categories within the aforementioned datasets, aggregating them based on their respective navigability profiles, as detailed in Table 2.

**Table 2:** Terrain navigability classification following Ref. [32].

Hierarchy level	RUGD Classes	RELLIS-3D Classes
Navigable-Smooth	concrete, asphalt	concrete, asphalt
Navigable-Rough	gravel, grass, dirt, sand, mulch	gravel, dirt
Navigable-Bumpy	rock, rock-bed	mud, rubble
Forbidden	water	water, puddle
Obstacles	tree, pole, log, etc.	tree, pole, log, etc.
Background	void, sky, sign	void, sky

It is important to emphasize that the adoption of navigability-based remapping is motivated by the fundamental functional requirements of autonomous mobility in unstructured terrains. In such stochastic environments, the primary objective of semantic segmentation often shifts from exhaustive taxonomic identification to the binary or hierarchical delineation of traversable versus non-traversable zones, which is paramount for real-time trajectory planning and operational safety. Furthermore, this reclassification facilitates a consistent benchmarking protocol between the RUGD and Rellis-3D datasets, effectively addressing the significant disjoint in their respective class ontologies and annotation schemas. While this aggregation concentrates the semantic scope on functional navigation, the inherent complexity of the task remains substantial due to the amorphous boundaries, geometric irregularities, and variable illumination typical of natural landscapes. Consequently, this approach ensures that the performance evaluation is directly aligned with the pragmatic exigencies of UGV deployment while maintaining a rigorous test of the model’s cross-modal alignment capabilities in the wild.

### 4.2. Evaluation metrics

To assess the efficacy of the proposed model, we adopt evaluation protocols consistent with prevailing segmentation benchmarks. Our quantitative analysis encompasses standard performance indicators, including Intersection over Union (*IoU*), Mean IoU (*mIoU*), Mean Pixel Accuracy (*mAcc*), and Average Pixel Accuracy (*aAcc*). In terms of formal notation, given an input visual field *I*, let *P*(*x*, *y*) denote the semantic label inferred at the spatial coordinate (*x*, *y*), while *G*(*x*, *y*) represents the associated ground truth category. Furthermore, 𝕀(X) serves as the indicator function, and *B* symbolizes the aggregate set of semantic class labels. The mathematical expressions governing these metrics are defined below:


$$\begin{array}{c} IoU_{i}=\frac{\sum_{I}\sum_{x,y}𝕀(P(x,y)=i\text{ and }G(x,y)=i)}{\sum_{I}\sum_{x,y}𝕀(P(x,y)=i\text{ or }G(x,y)=i)} \end{array}$$ (11)

$$\begin{array}{c} mIoU=\frac{\sum_{i}mIoU_{i}}{\sum_{B}1} \end{array}$$ (12)

$$\begin{array}{c} mAcc=\frac{\sum_{i\in B}\left(\sum_{I}\sum_{x,y,G(x,y)=i}𝕀(P(x,y)=G(x,y))\right)}{\sum_{B}1} \end{array}$$ (13)

$$\begin{array}{c} aAcc=\frac{\sum_{I}\sum_{x,y}𝕀(P(x,y)=G(x,y))}{\sum_{I}\sum_{x,y}1} \end{array}$$ (14)

### 4.3. Implementation details

The practical realization of the proposed framework relies on the MMSegmentation open-source toolkit [53], deployed atop the PyTorch version 1.10.1 environment. The backbone architecture leverages the pre-trained EVA-CLIP-1B [12], with all computational processes executed on a workstation featuring an RTX 5090 GPU. Regarding input specifications, the batch size is fixed at 2, while input imagery undergoes spatial cropping to resolutions of 300×375. The training phase on the RUGD dataset persists for a duration of 400K iterations. For optimization, we employ the AdamW algorithm, adhering to the default learning schedule protocols inherent to the MMSegmentation suite.

To ensure reproducibility, all experiments were conducted using a fixed random seed 42 to minimize stochastic variance. Preliminary tests indicated that *mIoU* fluctuated by less than 0.3% across multiple trials, suggesting low seed sensitivity. The learnable tokens for DPT were initialized using a truncated Gaussian distribution (μ=0,σ=0.02) to facilitate stable convergence from the onset of training. The total compute cost for the 400K iteration training regimen on an RTX 5090 GPU was approximately 52 hours, with an average inference latency of 45ms per frame. Training stability was monitored via the non-mutually exclusive loss function, which exhibited a smooth, monotonic decay, reaching a stable plateau after approximately 320K iterations.

### 4.4. Ablation study

In this subsection, we initiate the empirical analysis by gauging the segmentation precision of our proposed architecture on the RUGD repository. To explicitly disentangle the individual impacts of our core architectural components—namely DPT, EPE, and TIMO—on the overall performance uplift, a systematic ablation study is executed. Upon the conclusion of this internal assessment, the model, having been optimized solely on RUGD, is evaluated against the completely unobserved Rellis-3D dataset to strictly verify its cross-domain zero-shot generalization potential.

#### 4.4.1. Base model.

To substantiate the individual efficacy of the three proposed technical innovations, establishing a foundational baseline is imperative. Structurally, this reference model mirrors the general architectural schema of our proposed framework but is distinguished by specific exclusions. First, regarding the visual modality, the DPT mechanism is absent. Consequently, image feature extraction relies exclusively on the frozen EVA-CLIP encoder. Second, concerning the linguistic component, the complex prompt engineering is replaced by a singular, rudimentary template formulated as “*This is a photo of a {class}*.” to synthesize textual embeddings. Third, the TIMO module is omitted, with the system reverting to the standard ATM decoder for output generation. A comprehensive quantitative analysis, delineating performance variances across diverse configurations and combinations of these techniques on the RUGD benchmark, is presented in Table 3.

**Table 3:** Semantic segmentation performance on RUGD dataset with different combinations of proposed techniques under navigability analysis.

Model	*mIoU*	*mAcc*	*aAcc*
Baseline	72.22	82.65	90.68
Baseline + EPE	74.33	84.43	91.77
Baseline + DPT	77.52	88.76	94.87
Baseline + TIMO	81.45	91.02	95.58
Baseline + DPT + EPE	79.09	88.94	95.17
Baseline + EPE + TIMO	83.64	91.32	95.66
Baseline + DPT + TIMO	86.10	92.25	96.08
Baseline + EPE + DPT + TIMO	88.04	92.95	96.88

#### 4.4.2. Accuracy evaluation.

In this subsection, we dissect the isolated contributions of EPE, DPT, and TIMO toward the enhancement of semantic segmentation indices. Subsequently, a comparative benchmarking against contemporary state-of-the-art frameworks is presented to substantiate the superiority of our proposed architecture.

**(a) Effect of EPE**

Quantitative evidence from Table 3 reveals that the integration of EPE catalyzes a performance improvement of 2.1%, 1.8%, and 1.1% in *mIoU*, *mAcc*, and *aAcc*, respectively, relative to the baseline configuration. The advantage of EPE over monolithic text templates in this segmentation task stems from its capacity to assimilate multifaceted perspectives, thereby attenuating bias and broadening semantic coverage. By synthesizing a consensus from diverse prompts, the method achieves robustness against data fluctuations and error correction capabilities that a singular model cannot replicate. By harnessing this ensemble-based semantic consensus, EPE delivers a more holistic, precise, and resilient solution, adept at handling the heterogeneous input scenarios characteristic of unstructured environments.

**(b) Effect of DPT**

**Fig 5:**
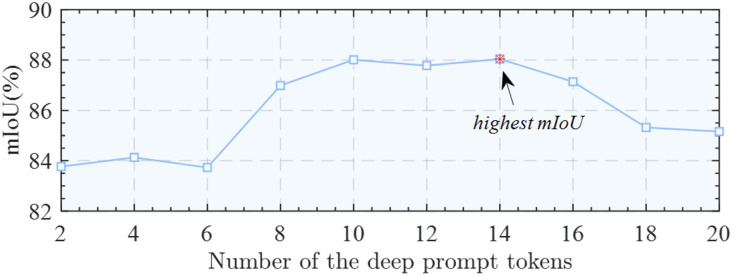
Influence of the number of deep tokens on *mIoU*.

Referring again to Table 3, the deployment of DPT induces notable gains, elevating *mIoU*, *mAcc*, and *aAcc* by 5.3%, 6.1%, and 4.2%, respectively, compared to the baseline. Crucially, the specific quantity of deep prompt tokens constitutes a pivotal hyperparameter governing the ultimate segmentation efficacy. The trajectory of *mIoU* performance as a function of token count is delineated in Fig 5. Analysis of this curve identifies a configuration of 14 tokens as the optimal setting for the RUGD testing set. Consequently, this value is adopted as the default standard throughout this study. Furthermore, it is observed that inflating the token count beyond this inflection point precipitates an adverse effect on model performance. This phenomenon is attributed to the necessity of aligning the volume of learnable tokens with the dataset’s magnitude and the intrinsic intricacy of the pixel-wise classification objective. For the specific demands of the RUGD dataset, a capacity of 14 tokens proves sufficient.

Beyond the mere quantity of tokens, the insertion depth of the DPT mechanism acts as a pivotal determinant of semantic segmentation efficacy. The impact of embedding learnable prompts at distinct hierarchical levels within the EVA-CLIP vision encoder is delineated in Table 4. Structurally, the EVA-CLIP vision encoder comprises a stack of 15 ViT layers, indexed sequentially from 1 (the foundational layer) to 15 (the uppermost layer). The precise insertion strata for these prompts are specified in the first column of Table 4. Empirical observation indicates that introducing prompt tokens into the lower layer yields superior efficacy compared to their deployment in higher layer. This trend is substantiated by the monotonic enhancement in evaluation metrics as the DPT depth configuration shifts from 10 → 15–5 → 10, and subsequently to 1 → 5. Furthermore, the maximal segmentation fidelity is realized by distributing learnable tokens throughout the entire depth of the ViT structure (layer = 1 → 15). Consequently, this ubiquitous configuration is adopted as the standard protocol for our experimental framework.

**Table 4:** Influence of the DPT depth on segmentation performance for RUGD dataset.

Layer	*mIoU*	*mAcc*	*aAcc*
1	85.23	87.98	92.07
1 → 5	87.64	90.76	93.13
1 → 10	87.92	91.33	94.22
1 → 15	88.04	92.95	96.88
5 → 10	85.95	88.65	92.49
5 → 15	86.34	89.74	92.86
10 → 15	85.48	88.12	92.27

**(c) Effect of TIMO**

This manuscript presents a core architectural innovation designated as TIMO, the theoretical underpinnings of which are elaborated in Subsection 3.3. Functionally, this module utilizes an attention-driven fusion strategy. It synthesizes the queried textual embeddings 𝒢 with the global visual descriptor [CLS] token $g^{l}$, derived from the EVA-CLIP backbone, to formulate a nuanced differential representation of the text-image pair. The assimilation of queries tailored to specific visual contexts within this architecture has been demonstrated to yield substantial improvements in segmentation fidelity. As corroborated by the quantitative data in Table 3, the deployment of TIMO precipitates marked enhancements over the baseline model, elevating the metrics of *mIoU*, *mAcc*, and *aAcc* by margins of 9.2%, 8.4%, and 4.9%, respectively. Such empirical outcomes are theoretically coherent, given the attention mechanism’s intrinsic proficiency in modeling the complex relational dependencies bridging the visual and linguistic encoding spaces.

**(d) Comparison with state-of-the-art methods**

Table 5 benchmarks the proposed methodology against a spectrum of prevailing state-of-the-art paradigms using the RUGD dataset, specifically focusing on the six distinct navigable categories. The quantitative analysis underscores that our architectural framework delivers a marked performance dividend, realizing an augmentation in *mIoU* that spans a margin of 1.2% to 43.9% relative to comparative techniques. In the context of the ten segmentation strategies evaluated, our approach consistently secures a position within the upper quintile (top 5) across the entire spectrum of navigable stratifications. Furthermore, it establishes supremacy by achieving the highest performance metrics in fifty percent of the designated segmentation levels.

**Table 5:** Comparison with state-of-the-art methods on RUGD dataset.

Methods	Smooth Region	Rough Region	Bumpy Region	Forbidden Region	Obstacle	Background	*mIoU*	*aAcc*
SegNeXt [54]	2.26	81.47	8.69	15.00	82.54	74.86	44.14	88.81
BiseNetv2 [55]	24.27	89.99	**89.99**	83.31	90.93	75.29	75.10	93.40
SDN [56]	83.03	92.82	87.69	81.05	90.94	75.11	85.11	94.77
GSS [57]	26.27	89.85	85.95	**84.13**	91.23	75.63	75.51	93.46
ContextFormer [58]	89.77	92.46	84.58	70.33	89.55	70.47	82.86	94.09
SCTNet [59]	1.04	81.23	22.98	25.84	89.18	74.50	49.13	88.77
PaSeNet [60]	**93.26**	93.16	87.56	77.31	91.20	78.50	86.83	95.17
SFMSegNet [61]	90.39	91.17	83.96	65.43	87.80	68.17	81.15	93.22
OffSeg [62]	92.76	93.28	87.44	79.90	89.55	66.46	84.90	94.24
Proposed method	90.27	**95.92**	86.88	77.64	**94.86**	**82.69**	**88.04**	**96.88**
Rank	4	1	2	5	1	1	1	1

#### 4.4.3 Cross-domain zero-shot ability evaluation.

To substantiate the cross-domain zero-shot generalization potential inherent in our methodology, Table 6 delineates a comparative evaluation against competing state-of-the-art architectures using the Rellis-3D benchmark. Crucially, it must be highlighted that within the context of Table 6, the comparative baselines were optimized via supervised learning exclusively on the Rellis-3D dataset. In stark contrast, our proposed framework relies solely on knowledge transfer from the RUGD dataset without exposure to Rellis-3D training samples. Consequently, these alternative methodologies function as a theoretical ceiling, effectively quantifying the performance gap between fully supervised learning and cross-domain zero-shot inference paradigms. Empirical evidence suggests that, with the exception of the background category, the proposed strategy yields outcomes that approach the performance of fully fine-tuned paradigms within the specific context of navigability-based classification. Specifically, it surpasses the majority of reference methods in the segmentation of navigable terrain classes and secures the 5^*th*^ rank in terms of the overall *mIoU* metric. Acknowledging the peripheral relevance of background elements to the core task of UGV mobility, and noting that despite occupying the penultimate ranking among the ten evaluated models, the system still attains a robust *mIoU* of 88.63% for the background class, it can be deduced that the framework is well-suited for autonomous navigation in heterogeneous unstructured domains. This adaptability is fundamentally attributed to the intrinsic cross-modal robustness provided by the EVA-CLIP architecture.

**Table 6:** Comparison with state-of-the-art methods on Rellis-3D dataset.

Methods	Smooth Region	Rough Region	Bumpy Region	Forbidden Region	Obstacle	Background	*mIoU*	*aAcc*
SegNeXt [54]	72.93	85.18	13.10	60.60	70.53	**95.95**	66.38	89.11
BiseNetv2 [55]	65.56	73.24	39.35	48.17	**71.91**	93.78	65.33	83.03
SDN [56]	67.06	77.60	**56.49**	49.76	70.31	94.43	69.27	84.51
GSS [57]	70.51	79.15	49.72	51.37	63.9	94.82	68.24	84.10
ContextFormer [58]	65.37	78.64	40.89	52.59	63.8	91.87	65.53	83.59
SCTNet [59]	5.42	76.65	47.13	54.87	62.74	85.50	55.38	81.61
PaSeNet [60]	60.28	79.78	53.35	53.78	70.15	94.37	68.62	85.37
SFMSegNet [61]	51.67	78.4	19.38	42.61	66.04	92.05	58.36	82.16
OffSeg [62]	**76.73**	**86.86**	23.49	**71.58**	71.24	94.65	**70.76**	**90.44**
Proposed method	71.48	80.32	43.64	53.28	69.99	88.63	67.89	85.47
Rank	3	3	5	5	6	9	5	3

**Fig 6:**
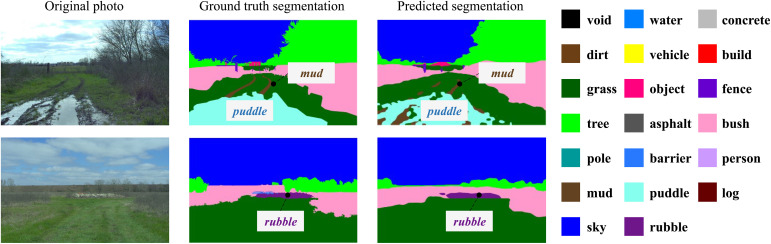
Cross-domain zero-shot capability of the proposed method on unseen classes.

Furthermore, a scrutiny of Table 1 reveals a disjoint in class ontology, wherein the Rellis-3D corpus includes semantic categories that are conspicuously absent from the RUGD archive. As a direct consequence, conventional supervised segmentation frameworks, when restricted to training on RUGD data, are fundamentally incapable of recognizing these disparate categories. Conversely, by harnessing the intrinsic cross-modal alignment properties of the EVA-CLIP architecture, our proposed framework demonstrates the capacity to extrapolate accurate segmentation masks for these novel categories within the Rellis-3D environment, despite their total exclusion from the training phase. Visual substantiation is provided in Fig 6, which depicts qualitative results where the system exhibits robust efficacy in delineating previously unseen entities, specifically identifying *puddle*, *rubble*, and *mud*.

## 5. Conclusion and future work

This study elucidates a novel cross-domain zero-shot framework tailored for semantic segmentation in non-geometric terrains, essentially augmenting the cross-modal alignment proficiency of the EVA-CLIP architecture. The methodology strategically integrates DPT to refine the model’s contextual flexibility, alongside EPE to optimize segmentation fidelity. Through the synergistic fusion of global and local feature representations, the system significantly fortifies text-image correspondence, thereby eclipsing contemporary techniques with an appreciable elevation in *mIoU* ranging from 1.2% to 43.9% on the RUGD benchmark. Furthermore, the approach exhibits efficacy commensurate with supervised fine-tuning protocols on the Rellis-3D dataset, indicating robust generalization capabilities regarding unobserved semantic categories.

While our results demonstrate a robust cross-domain zero-shot foundation, the observed generalization efficacy is currently constrained by a single-source transfer from RUGD to Rellis-3D using remapped navigability profiles. As these findings do not yet constitute a universal equivalence to supervised learning across all unstructured domains, future research will prioritize broader multi-domain evaluations—encompassing diverse geographic locales and granular class ontologies—to definitively establish the model’s comprehensive transferability. Moreover, to bridge the gap between theoretical design and pragmatic implementation, we intend to deploy the framework onto physical UGV platforms to substantiate its utility in operational settings. The subsequent integration with downstream path planning and motion control will culminate in a unified navigation architecture, providing the essential empirical validation required for cultivating resilient and versatile autonomous systems in complex environments.

## References

[1] Fan J, Wang Z, Geng J, Chen Y, Jiang Y, Zhang X. Probabilistic traversability risk-aware one-stage motion planner for unmanned ground vehicle in unstructured environments. In: 2025 IEEE 102nd Vehicular Technology Conference (VTC2025-Fall), 2025. 1–6. 10.1109/vtc2025-fall65116.2025.11310121

[2] Hu J, Chen J, Lv M, Xu Z, Chen Z, Han J. MSHF: Multi-sensor hierarchical fusion for UGV localization in unstructured environment. Expert Systems with Applications. 2025;294:128732. doi: 10.1016/j.eswa.2025.128732

[3] Fan J, Zhang X, Zheng K, Zou Y, Zhou N. Hierarchical path planner combining probabilistic roadmap and deep deterministic policy gradient for unmanned ground vehicles with non-holonomic constraints. Journal of the Franklin Institute. 2024;361(8):106821. doi: 10.1016/j.jfranklin.2024.106821

[4] Feng Y, Li X, Ni P, Liu X, Jiang T. Multisensor Fusion Network for Unstructured Scene Segmentation With Surface Normal Incorporated. IEEE Sensors J. 2024;24(8):13589–603. doi: 10.1109/jsen.2024.3370255

[5] Fan J, Zhang X, Chen Y, Zou Y, Du G, Li Y, et al. Vision-Based Local Motion Planner in Unstructured Environments Using Semantic Segmentation Guided Traversability Modeling and Parallelized Model Predictive Path Integral Algorithm. Automot Innov. 2026. doi: 10.1007/s42154-025-00394-4

[6] Li J, Xie Y, Huo Y. An Independent Suspension and Trafficability Analysis for an Unmanned Ground Platform. Symmetry. 2025;17(1):128. doi: 10.3390/sym17010128

[7] Baheti B, Innani S, Gajre S, Talbar S. Eff-UNet: A Novel Architecture for Semantic Segmentation in Unstructured Environment. In: 2020 IEEE/CVF Conference on Computer Vision and Pattern Recognition Workshops (CVPRW), 2020. 1473–81. 10.1109/cvprw50498.2020.00187

[8] Liu Y, Wang S, Wang C, Lu M, Sang Y. Latent domain knowledge distillation for nighttime semantic segmentation. Engineering Applications of Artificial Intelligence. 2024;132:107940. doi: 10.1016/j.engappai.2024.107940

[9] Fan J, Zhang X, Zou Y, Li Y, Liu Y, Sun W. Improving policy training for autonomous driving through randomized ensembled double Q-learning with Transformer encoder feature evaluation. Applied Soft Computing. 2024;167:112386. doi: 10.1016/j.asoc.2024.112386

[10] Muhammad K, Hussain T, Ullah H, Ser JD, Rezaei M, Kumar N, et al. Vision-Based Semantic Segmentation in Scene Understanding for Autonomous Driving: Recent Achievements, Challenges, and Outlooks. IEEE Trans Intell Transport Syst. 2022;23(12):22694–715. doi: 10.1109/tits.2022.3207665

[11] Blumenstiel B, Jakubik J, Kuehne H, Vössing M. What a MESS: Multi-Domain Evaluation of Zero-Shot Semantic Segmentation. In: Advances in Neural Information Processing Systems 36, 2023. 73299–311. 10.52202/075280-3205

[12] Radford A, Kim JW, Hallacy C, Ramesh A, Goh G, Agarwal S. In: International conference on machine learning, 2021. 8748–63.

[13] Deng W, Gedeon T, Tu W. A Closer Look at the Robustness of Contrastive Language-Image Pre-Training (CLIP). In: Advances in Neural Information Processing Systems 36, 2023. 13678–91. 10.52202/075280-0603

[14] Hu X, Zhang K, Xia L, Chen A, Luo J, Sun Y, et al. ReCLIP: Refine Contrastive Language Image Pre-Training with Source Free Domain Adaptation. In: 2024 IEEE/CVF Winter Conference on Applications of Computer Vision (WACV), 2024. 2982–91. 10.1109/wacv57701.2024.00297

[15] Sun Q, Wang J, Yu Q, Cui Y, Zhang F, Zhang X. Eva-clip-18b: Scaling clip to 18 billion parameters. In: 2024. https://doi.org/arXiv:240204252

[16] Allingham JU, Ren J, Dusenberry MW, Gu X, Cui Y, Tran D, et al. In: International Conference on Machine Learning, 2023. 547–68.

[17] Sohn K, Chang H, Lezama J, Polania L, Zhang H, Hao Y, et al. Visual Prompt Tuning for Generative Transfer Learning. In: 2023 IEEE/CVF Conference on Computer Vision and Pattern Recognition (CVPR), 2023. 19840–51. 10.1109/cvpr52729.2023.01900

[18] Emek Soylu B, Guzel MS, Bostanci GE, Ekinci F, Asuroglu T, Acici K. Deep-Learning-Based Approaches for Semantic Segmentation of Natural Scene Images: A Review. Electronics. 2023;12(12):2730. doi: 10.3390/electronics12122730

[19] Fooladgar F, Kasaei S. A survey on indoor RGB-D semantic segmentation: from hand-crafted features to deep convolutional neural networks. Multimed Tools Appl. 2019;79(7–8):4499–524. doi: 10.1007/s11042-019-7684-3

[20] Long J, Shelhamer E, Darrell T. Fully convolutional networks for semantic segmentation. In: 2015 IEEE Conference on Computer Vision and Pattern Recognition (CVPR), 2015. 3431–40. 10.1109/cvpr.2015.7298965 27244717

[21] Je C, Jeon HS, Son C-H, Park H-M. Disparity-based space-variant image deblurring. Signal Processing: Image Communication. 2013;28(7):792–808. doi: 10.1016/j.image.2013.04.005

[22] Du G, Cao X, Liang J, Chen X, Zhan Y. Medical image segmentation based on U-Net: A review. Journal of Imaging Science & Technology. 2020;64(2).

[23] Chen L-C, Zhu Y, Papandreou G, Schroff F, Adam H. Encoder-Decoder with Atrous Separable Convolution for Semantic Image Segmentation. Lecture Notes in Computer Science. Springer International Publishing. 2018. p. 833–51. 10.1007/978-3-030-01234-2_49

[24] Fu J, Liu J, Tian H, Li Y, Bao Y, Fang Z, et al. Dual Attention Network for Scene Segmentation. In: 2019 IEEE/CVF Conference on Computer Vision and Pattern Recognition (CVPR), 2019. 3141–9. 10.1109/cvpr.2019.00326

[25] Xie E, Wang W, Yu Z, Anandkumar A, Alvarez JM, Luo P. SegFormer: Simple and efficient design for semantic segmentation with transformers. Advances in Neural Information Processing Systems. 2021;34:12077–90.

[26] Strudel R, Garcia R, Laptev I, Schmid C. Segmenter: Transformer for Semantic Segmentation. In: 2021 IEEE/CVF International Conference on Computer Vision (ICCV), 2021. 7242–52. 10.1109/iccv48922.2021.00717

[27] Ranftl R, Bochkovskiy A, Koltun V. Vision Transformers for Dense Prediction. In: 2021 IEEE/CVF International Conference on Computer Vision (ICCV), 2021. 12159–68. 10.1109/iccv48922.2021.01196

[28] Das A, Xian Y, He Y, Akata Z, Schiele B. Urban Scene Semantic Segmentation with Low-Cost Coarse Annotation. In: 2023 IEEE/CVF Winter Conference on Applications of Computer Vision (WACV), 2023. 5967–76. 10.1109/wacv56688.2023.00592

[29] Beycimen S, Ignatyev D, Zolotas A. A comprehensive survey of unmanned ground vehicle terrain traversability for unstructured environments and sensor technology insights. Engineering Science and Technology, an International Journal. 2023;47:101457. doi: 10.1016/j.jestch.2023.101457

[30] Khairnar S, Thepade SD, Kolekar S, Gite S, Pradhan B, Alamri A, et al. Enhancing semantic segmentation for autonomous vehicle scene understanding in indian context using modified CANet model. MethodsX. 2024;14:103131. doi: 10.1016/j.mex.2024.103131 39846010 PMC11751566

[31] Kumar GA, Mohiddin MdK, Mishra SK, Verma A, Sharma M, Naresh A. Enhancing Autonomous Vehicle Navigation in Complex Environment With Semantic Proto‐Reinforcement Learning. Journal of Field Robotics. 2025;42(5):2042–61. doi: 10.1002/rob.22506

[32] Guan T, Kothandaraman D, Chandra R, Sathyamoorthy AJ, Weerakoon K, Manocha D. GA-Nav: Efficient Terrain Segmentation for Robot Navigation in Unstructured Outdoor Environments. IEEE Robot Autom Lett. 2022;7(3):8138–45. doi: 10.1109/lra.2022.3187278

[33] Lin N, Zhao W, Liang S, Zhong M. Real-Time Segmentation of Unstructured Environments by Combining Domain Generalization and Attention Mechanisms. Sensors (Basel). 2023;23(13):6008. doi: 10.3390/s23136008 37447855 PMC10346488

[34] Fu Y, Gao M, Xie G, Hu M, Wei C, Ding R. Density-Aware U-Net for Unstructured Environment Dust Segmentation. IEEE Sensors J. 2024;24(6):8210–26. doi: 10.1109/jsen.2024.3355388

[35] Chen N, Wei D, Lin D, Lin L. Semantic SLAM using laser-vision data fusion: Enhancing autonomous navigation in unstructured environments. Alexandria Engineering Journal. 2025;127:606–18. doi: 10.1016/j.aej.2025.05.015

[36] Wang Y, Tian Y. Exploring zero-shot semantic segmentation with no supervision leakage. Electronics. 2023;12(16). doi: 10.3390/electronics12163452

[37] Radford A, Wu J, Child R, Luan D, Amodei D, Sutskever I. Language models are unsupervised multitask learners. OpenAI blog. 2019.

[38] Guo S-C, Liu S-K, Wang J-Y, Zheng W-M, Jiang C-Y. CLIP-Driven Prototype Network for Few-Shot Semantic Segmentation. Entropy (Basel). 2023;25(9):1353. doi: 10.3390/e25091353 37761652 PMC10529322

[39] Ali M, Khan S. CLIP-Decoder : ZeroShot Multilabel Classification using Multimodal CLIP Aligned Representations. In: 2023 IEEE/CVF International Conference on Computer Vision Workshops (ICCVW), 2023. 4677–81. 10.1109/iccvw60793.2023.00505

[40] Zhou Z, Lei Y, Zhang B, Liu L, Liu Y. ZegCLIP: Towards Adapting CLIP for Zero-shot Semantic Segmentation. In: 2023 IEEE/CVF Conference on Computer Vision and Pattern Recognition (CVPR), 2023. 11175–85. 10.1109/cvpr52729.2023.01075

[41] Meng Q, Gao Y, He Y, Hussain S, Lu J, Chen Y, et al. PowerMistral: A data-efficient wind power forecasting framework leveraging pre-trained large language models. Applied Energy. 2026;411:127625. doi: 10.1016/j.apenergy.2026.127625

[42] Liu J, Wang Z, Zang X, Li X, Guo L, Meng Q. Data-driven dynamic assessment of wind farm frequency characteristics based on state space mapping. CSEE Journal of Power and Energy Systems. 2025;11(3):1018–29. doi: 10.17775/CSEEJPES.2023.02430

[43] Gao Y, Tahir M, Siano P, Hussain S, Sun W, He Y, et al. A bi-level hybrid game framework for Stochastic Robust optimization in multi-integrated energy microgrids. Sustainable Energy, Grids and Networks. 2025;44:102024. doi: 10.1016/j.segan.2025.102024

[44] Sennrich R. Neural machine translation of rare words with subword units. arXiv preprint. 2015. https://arxiv.org/abs/1508.07909

[45] Chu X, Liu Y, Shen C, Tang Q, Tian Z, Wei X, et al. SegViT: Semantic Segmentation with Plain Vision Transformers. In: Advances in Neural Information Processing Systems 35, 2022. 4971–82. 10.52202/068431-0359

[46] Everingham M, Eslami SMA, Van Gool L, Williams CKI, Winn J, Zisserman A. The Pascal Visual Object Classes Challenge: A Retrospective. Int J Comput Vis. 2014;111(1):98–136. doi: 10.1007/s11263-014-0733-5

[47] Lin T-Y, Maire M, Belongie S, Hays J, Perona P, Ramanan D, et al. Microsoft COCO: Common Objects in Context. Lecture Notes in Computer Science. Springer International Publishing. 2014. p. 740–55. 10.1007/978-3-319-10602-1_48

[48] Zhou B, Zhao H, Puig X, Fidler S, Barriuso A, Torralba A. Scene Parsing through ADE20K Dataset. In: 2017 IEEE Conference on Computer Vision and Pattern Recognition (CVPR), 2017. 5122–30. 10.1109/cvpr.2017.544

[49] Cordts M, Omran M, Ramos S, Rehfeld T, Enzweiler M, Benenson R, et al. The Cityscapes Dataset for Semantic Urban Scene Understanding. In: 2016 IEEE Conference on Computer Vision and Pattern Recognition (CVPR), 2016. 3213–23. 10.1109/cvpr.2016.350

[50] Geiger A, Lenz P, Stiller C, Urtasun R. Vision meets robotics: The KITTI dataset. The International Journal of Robotics Research. 2013;32(11):1231–7. doi: 10.1177/0278364913491297

[51] Wigness M, Eum S, Rogers JG, Han D, Kwon H. A RUGD Dataset for Autonomous Navigation and Visual Perception in Unstructured Outdoor Environments. In: 2019 IEEE/RSJ International Conference on Intelligent Robots and Systems (IROS), 2019. 5000–7. 10.1109/iros40897.2019.8968283

[52] Jiang P, Osteen P, Wigness M, Saripalli S. RELLIS-3D Dataset: Data, Benchmarks and Analysis. In: 2021 IEEE International Conference on Robotics and Automation (ICRA), 2021. 1110–6.

[53] Contributors M. MMSegmentation: Openmmlab semantic segmentation toolbox and benchmark; 2020.

[54] Guo MH, Lu CZ, Hou Q, Liu Z, Cheng MM, Hu SM. SegNeXt: Rethinking Convolutional Attention Design for Semantic Segmentation. 2022 https://arxiv.org/abs/2209.08575

[55] Yu C, Gao C, Wang J, Yu G, Shen C, Sang N. Bisenet v2: Bilateral network with guided aggregation for real-time semantic segmentation. International journal of computer vision. 2021;129:3051–3068.

[56] Tan H, Wu S, Pi J. Semantic Diffusion Network for Semantic Segmentation; 2023 https://arxiv.org/abs/2302.02057

[57] Chen J, Lu J, Zhu X, Zhang L. Generative Semantic Segmentation; 2023. https://arxiv.org/abs/2303.11316

[58] Abid MMN, Mehta N, Wu Z, Timofte R. ContextFormer: Redefining Efficiency in Semantic Segmentation; 2025 https://arxiv.org/abs/2501.19255

[59] Xu Z, Wu D, Yu C, Chu X, Sang N, Gao C. SCTNet: Single-Branch CNN with Transformer Semantic Information for Real-Time Segmentation; 2024 https://arxiv.org/abs/2312.17071

[60] Chen G, Li H, Li Y, Zhang W, Song T. Parallel segmentation network for real-time semantic segmentation. Engineering Applications of Artificial Intelligence. 2025;148:110487. doi: 10.1016/j.engappai.2025.110487

[61] Chen L, Fu Y, Gu L, Zheng D, Dai J. Spatial Frequency Modulation for Semantic Segmentation; 2025. https://arxiv.org/abs/2507.11893 10.1109/TPAMI.2025.3592621 40705587

[62] Zhang SC, Li Y, Wu YH, Hou Q, Cheng MM. Revisiting Efficient Semantic Segmentation: Learning Offsets for Better Spatial and Class Feature Alignment. 2025. https://arxiv.org/abs/2508.08811

